# Depression and Vitamin D: A Peculiar Relationship

**DOI:** 10.7759/cureus.24363

**Published:** 2022-04-21

**Authors:** Nisha Saji Parel, Parimi Vamsi Krishna, Anuradha Gupta, Kamsika Uthayaseelan, Kivonika Uthayaseelan, Monika Kadari, Muhammad Subhan, Sripada Preetham Kasire

**Affiliations:** 1 Family Medicine, Tbilisi State Medical University, Tbilisi, GEO; 2 Internal Medicine, Jagadguru Jayadeva Murugarajendra Medical College, Davanagere, IND; 3 Research, Government Medical College Nagpur, Nagpur, IND; 4 Internal Medicine, All Saints University College of Medicine, Saint Vincent and the Grenadines, Kingstown, VCT; 5 Internal Medicine, All Saints University School of Medicine, Dominica, Roseau, DMA; 6 Internal Medicine, Bhaskar Medical College, Hyderabad, IND; 7 Internal Medicine, Jinnah Hospital, Lahore - Allama Iqbal Medical College, Lahore, PAK; 8 Internal Medicine, Mamata Medical College, Hyderabad, IND

**Keywords:** age and depression, serum vitamin d levels, serum 25-hydroxy vitamin d level, depression in elderly, post partum depression, depression, depression prevention, vitamin d supplementation, vitamin-d deficiency, vitamin d & depression

## Abstract

Depression is a psychiatric disorder characterized by various symptoms that can impact one's quality of life. Vitamin D, a fat-soluble vitamin, is well-known for its role in bone health, and research on its effects on mental health has only recently emerged. Vitamin D deficiency is widespread worldwide, and it has been linked to an increased risk of depression. In this article, we have discussed different hypotheses that explain the role of vitamin D in gene expression and its effects on neurotransmitters and different brain functions. We have reviewed literature that shows us that Vitamin D deficiency is a risk factor for depression and explored studies that show us the effects of using or supplementing Vitamin D in preventing depression among various populations.

## Introduction and background

Major depressive disorder (MDD) is a psychiatric illness marked by various symptoms that can harm one's quality of life [1]. The earliest recorded reports of what is now considered depression were seen in the second millennium B.C.E in Mesopotamia, where depression was considered a spiritual affliction rather than a mental disorder [2]. During the 1970s, physicians in the United States coined the term major depressive disorder [3]. In 1980, it officially became a part of the Diagnostic and Statistical Manual of Mental Disorders-III (DSM) [3]. In 2008, WHO ranked MDD as the third cause of burden of disease and has estimated that by 2030 this disorder will rank first [4]. MDD is two times more prevalent in women than men [5]. People with comorbid psychiatric disorders such as social anxiety and panic disorders or substance use have a higher risk of suicide when diagnosed with MDD [6]. Genetic and environmental factors contribute to the etiology of depression, and some studies report that genetic factors mainly play a role in early-onset depression [7]. In the elderly, strokes, seizure disorders, neurodegenerative diseases, and chronic pain have been implicated with greater risk for developing depression [7]. Other environmental factors such as financial problems, traumatic experiences, death of a loved one, conflicts, and lack of social support are other examples that could trigger depression in an individual [7]. The underlying pathophysiology has not been clearly understood; however, earlier evidence shows that there are abnormalities in neurotransmitters like serotonin (5-HT), norepinephrine (NE), dopamine (DA), glutamate, and brain derived neurotrophic factor (BDNF) [7]. The multifactorial reasons contributing to depression can cause modifications in neuroendocrine and behavioral reactions, which can cause functional and anatomical alterations such as enhanced hyperintensities in subcortical areas and decreased anterior brain metabolism on the left side, respectively [6].

The following is a list of the DSM-5's nine symptoms (Table 1) [7]. To make a clinical diagnosis, five symptoms out of the nine must be present (one of which should be a sad mood or anhedonia):

**Table 1:** Clinical features of depression DSM-5 - Diagnostic and Statistical Manual of Mental Disorders, Fifth Edition

Sl. no.	DSM-5 Criteria
1.	Feeling sad/low
2.	Anhedonia
3.	Thoughts of unworthiness/guilt
4.	Changes in energy/fatigue
5.	Psychomotor retardation or agitation
6.	Difficulty concentrating
7.	Changes in appetite/weight
8.	Thoughts of suicide/death
9.	Difficulty sleeping

Depression can be managed by different treatment approaches, which include lifestyle modification, pharmacological (such as selective serotonin reuptake inhibitors (SSRIs), serotonin/norepinephrine reuptake inhibitors (SNRIs), atypical antidepressants, serotonin-dopamine activity modulators (SDAMs), tricyclic antidepressants (TCAs), monoamine oxidase inhibitors (MAOIs)) [8-13], interventional and psychotherapeutic approaches (such as cognitive behavioral therapy) [6], and combining pharmacological and psychotherapeutic approaches have been proven to be more effective in treatment [14-16]. For severe major depression, electroconvulsive therapy has been more effective than any other therapeutic option [17]. The various treatment approaches to depression are summarized in Table 2.

**Table 2:** Summary of various approaches to management of depression SSRI- selective serotonin re-uptake inhibitor, 5HT- serotonin, SERT- serotonin transporter, NE- norepinephrine, SNRI- serotonin norepinephrine reuptake inhibitor, NET- norepinephrine transporter, TCA- tricyclic antidepressants. MAOI- monoamine oxidase inhibitor, SDAM- serotonin dopamine activity modulator, 5HT1A- serotonin type 1A receptor, D2- dopamine type 2 receptor, 5HT2A-serotonin type 2A receptor, CBT- cognitive behavioral therapy, ECT- electroconvulsive therapy

Types of treatment	Mechanisms
Lifestyle Modifications	Improving quality of sleep, exercise, healthy eating, meditation, stress management, avoiding alcohol/drug use
Classes of Medications	SSRI-inhibit reuptake of 5HT by binding to SERT; SNRI-inhibit reuptake of both serotonin and NE by binding to SERT and NET and weakly inhibits dopamine reuptake; TCA-inhibit reuptake of both 5HT and NE by binding to SERT and NET, antihistamine & anticholinergic; MAOI inhibits the activity of monoamine oxidase, thus preventing the breakdown of monoamine neurotransmitters and increasing their availability; SDAMs- partial agonist at 5-HT1A and D2 receptors and an inhibitor at 5-HT2A and NE alpha type 1B and type 2C receptors, with similar potencies; Atypicals - dopamine reuptake inhibitor, 5HT receptor modulator, inhibits alpha two receptors
Psychotherapy: CBT, Interpersonal and psychodynamic	It aids in the recognition and modification of negative thoughts and behaviours.
ECT	A seizure is triggered by electrical stimulation when used a few times a week for a short time and eliminates depression symptoms for a prolonged time.

Hypovitaminosis can lead to various conditions, some of which affect mental health [18]. An increasing number of studies link depression with vitamin D deficiency [19]. Since it is theorized that vitamin D plays a role in gene expression, it regulates the level of 5-HT, DA, and NE receptors in the brain, and low vitamin D levels result in a decrease of these neurotransmitters, which leads to depression [20-23]. This article aims to discuss the role and association of vitamin D in the pathophysiology and management of depression, highlight the impact of vitamin D deficiency and depression in different population groups, and look into the possibility of using vitamin D as an adjunct to antidepressants for a better prognosis.

## Review

Vitamin D, a fat-soluble vitamin, can be obtained from various sources, including sun exposure, regular dietary intake, and dietary pills [24]. Ultraviolet (UV) B radiation from sunlight enters the skin and transforms 7-dehydrocholesterol to previtamin D3, quickly transforming to vitamin D3 [25]. UV light irradiation produces vitamin D2 from yeast, while vitamin D3 is made by UV irradiation of 7-dehydrocholesterol from lanolin [24]. Vitamin D that is absorbed from the skin and dietary intake is converted in the liver to 25-hydroxyvitamin D (25(OH)D), which can be used to assess a patient's vitamin D level [25-28]. The kidneys then convert 25(OH)D by the enzyme 25(OH)D-1α-hydroxylase (CYP27B1) to its active form, 1,25-dihydroxy vitamin D [25-28]. It subsequently binds to vitamin D receptors in target organs to control gene transcription and cell membrane structures to conduct different non-genomic responses [29]. Vitamin D receptors are seen in almost every tissue and cell in the body [30,31]. In the brain, it can be found primarily on the hippocampus, prefrontal cortex, hypothalamus, cingulate gyrus, substantia nigra, and thalamus [30,31]. This is important because many of those brain areas have been linked to depression's physiology [32]. According to increasing research, vitamin D is a neuroactive steroid that plays a critical role in the expression of neurotransmitters with its regulation and neuroimmunomodulation, antioxidant production, and various neurotrophic factors, making it biochemically plausible that vitamin D is associated with depressive symptoms [33]. Although the mechanism by which vitamin D works in the body is unknown, a few hypotheses show us an association between these two [33]. 

According to the neurotrophic hypothesis, immunohistochemical research has discovered vitamin D receptors (VDRs) in the central nervous system (CNS), providing a solid indication that vitamin D plays a significant role in brain functions [33]. VDRs are found all over the brain, including the hippocampus, which plays a role in controlling memory and emotional function [33]. Because the hippocampus is vital in the causes of depression, discovering VDR within it has encouraged many researchers to investigate the effects of vitamin D on hippocampal shape and function in animals [34]. Numerous investigations using in vitro culturing of hippocampus cells and in vivo experiments on the brains of adult mice have revealed that vitamin D deficiency can alter the shape or function of the hippocampal development [33]. Croll et al. conducted a cross-sectional analysis on 2716 people in the Netherlands (from 2006 to 2009), which showed that those with vitamin D deficiency (serum concentration 30 nmol/L) had decreased brain tissue and hippocampal volume, as seen in their brain magnetic resonance imaging (MRI) [35].

Numerous studies have demonstrated that vitamin D is a potent regulator of the production of neurotrophic substances, such as BDNF, a neurotrophin (NT)-3, and nerve growth factor (NGF) [33]. Neurotrophic factors are necessary for neuron survival, development, and migration, in which they drive their physiological function by combining with their corresponding tropomyosin-related kinase (Trk) receptors, including BDNF/TrkB, NT-3/TrkC, NGF/TrkA, as well as the common p75 neurotrophin receptor (p75NTR) [36]. Various research has shown that 1,25(OH)2D can enhance the production of BDNF, NGF, and NT-3 and downregulate NT-4 in the astrocytes of the brain, indicating that vitamin D plays a role in neuronal survival and differentiation during the development [37,38]. In the adult hippocampus, BDNF is critical for the long-term viability, specialization, and performance of new neurons [33]. NT-3 and NT-4 are necessary for the survival of growing neurons and for the differentiation and proliferation of precursor cells, thereby directly or indirectly affecting the cause of depression [33]. As a result, vitamin D can influence neurotrophic agents, whose aberrant performance has been linked to various psychiatric disorders [33]. According to the classic monoamine neurotransmission hypothesis, the deficiency of monoamines such as 5-HT, DA, and NE can cause depression [33].

On the other hand, vitamin D deficiency may interfere with the synthesis of 5-HT, resulting in the aberrant development of serotonergic and brain neurons [33]. 5-HT also functions on the hippocampus, where the production of new neurons and synaptic plasticity have been linked to the onset and treatment of depression [33]. The VDR is found in dopaminergic neurons in the substantia nigra, prefrontal cortex, and hippocampus of humans and rats, all of which are linked to depression [39]. Vitamin D deficiency can cause a delay in DA cell differentiation because of its effects on VDR expression in the substantia nigra, which can lead to DA-mediated behavioral deficits [40]. It also suggests that vitamin D deficiency can affect dopaminergic neuron development and have severe effects on the evolution of depression [33]. Hence, vitamin D directly or indirectly influences the levels of monoamines in the body and is involved in the pathogenesis of depression [33].

Literature review of the association between vitamin D and depression

In Adults and Elderly

It is crucial to note that vitamin D deficiency has been seen in a range of populations, including children, adolescents, adults, and the elderly, irrespective of race, ethnicity, or nation [41-43]. Depression is related to cardiovascular (CV) events, and it has been indicated that vitamin D deficiency may be linked to depression and a significant contributor to a higher risk of CV events [44]. May et al. conducted a nine-year cohort study in Utah, USA, which found that lower vitamin D levels were significantly associated with depression among 7358 patients aged 50 years or older with cardiovascular disease [44]. In the elderly, vitamin D deficiency is a risk factor for developing depression [45]. Milaneschi et al. conducted a population-based cohort study in Tuscany, Italy, to explore the connection between 25(OH)D levels and depression symptoms in 423 men and 531 women aged 65 years and older over six years [45]. Men (hazard ratio = 1.6; 95 % confidence interval (CI) = 0.9-2.8; P = 0.1) and women (hazard ratio = 2.0; 95 % confidence interval (CI) = 1.2-3.2; P = 0.005) with a serum 25(OH)D of less than 50 nmol/L had a higher risk of depression, with the magnitude of the potential association being greater in women than in men [45]. The study mentioned above is similar to a cohort study by Chan et al. in Hong Kong, China, which included 939 males aged 65 and up [46]. The results demonstrated that there is an inverse relationship between serum 25(OH)D and depression (odds ratio = 0.46, 95 percent CI: 0.22-0.98, P=0.004) in 629 males after a four-year follow-up period [46]. A non-interventional prospective cohort study done by Lee et al. in Europe also concluded an inverse relationship between depression and 25(OH)D levels [47]. The study had included 3369 men, aged between 49 to 71 years, that were taking part in the European Male Ageing Study, depression was evaluated using the Beck Depression Inventory-II (BDI-II), and radioimmunoassay was used to assess serum 25(OH)D and parathyroid hormone (PTH) levels [47]. Its results showed decreased levels of 25(OH)D were associated with increased BDI-II score (p = 0.004) [47]. Late-life depression can also be linked to vitamin D deficiency [48]. According to Stewart and Hirani's data analyzed among 2070 people in England 65 years or older showed that depressive symptoms were related to vitamin D deficiency when the 25(OH)D levels were lower than 10ng/mL [48].

In Women and During Pregnancy

Vitamin D deficiency has traditionally been known to harm the bones, such as having osteopenia and osteoporosis due to having low bone mineral density (BMD) [49]. Premenopausal women with depression and older African-American adults with Vitamin D deficiency are more likely to have low BMD [49,50]. Depression is the most common mood illness in pregnancy and postpartum due to significant hormonal, physical, and social changes [51]. In developing countries, the occurrence of depression in pregnancy has been estimated to be as high as 20%, while it was between 10% and 15% in developed countries [52]. Moreover, 20-40 percent of women globally suffer from postpartum depression (PPD), which is defined as a nonpsychotic depressive disorder that occurs within a year following childbirth [53,54]. Lower levels of Vitamin D in pregnant women have resulted in increased depressive symptoms [55]. A study by Cassidy-Bushrow et al. in Detroit that assessed vitamin D levels and depression screening in 178 pregnant African American women showed that there was a strong inverse correlation between log (25-OHD) and Center for Epidemiologic Studies-Depression scale (CES-D) score of ≥16 [54]. The chances of CES-D ≥16 decreased by 46% for every 1-unit rise in log (25-OHD) (equivalent to 2.72 ng/mL increase in 25-(OH)D) (OR=0.54, 95% CI. 0.29-0.99, p=0.046) [55]. The above study results are comparable to a cohort study done in Amsterdam by Brandenbarg et al. among 4101 pregnant women whose vitamin D levels were assessed at 13 weeks gestation [56]. Screening for depression was done using the CES-D scale (score ≥ 16) at 16 weeks gestation, and the study concluded that early-pregnancy vitamin D deficiency was linked to increased depression symptoms during pregnancy [56]. Gur et al. conducted a cohort study in Turkey that included 179 pregnant women between 24 and 28 weeks of pregnancy [57]. Depression was screened for using The Edinburgh Postnatal Depression Scale (EPDS), and vitamin D levels were assessed [57]. The above study is similar to a cohort study done by Fu et al. in Beijing, China, where serum vitamin D levels were assessed 24-48 hours postpartum, and EPDS was used to screen for depression (score ≥12) three months postpartum [58]. The study also concluded that lower 25(OH)D levels were linked to PPD [58]. The above-mentioned studies [44-48, 54, 56-58] are summarized in Table 3 below:

**Table 3:** Summary of included studies linking depression and vitamin D deficiency BDI-II - Beck Depression Inventory-II, Vit D-Vitamin D, PTH- Parathyroid hormone, 25(OH)D-25 - Hydroxyvitamin D, ICD-9 - International Classification of Diseases, Ninth Revision, GDS- Geriatric Depression Scale, VDD- Vitamin D Deficiency, CES-D - Center for Epidemiological Studies-Depression Scale, EPDS- Edinburgh Postnatal Depression Scale, PPD- Postpartum depression, NA- not available

References	Type of study	Sample size	Population	Location	Diagnostic criteria	Conclusion
May et al. (2010) [44]	COHORT	7358	Adults aged 50 years or older with CVD	UTAH, USA	Depression- ICD-9 Vit D- >50, 31-50,16-30 and or = 15	Vit D levels were significantly associated with depression .
Milaneschi et al (2010) [45]	COHORT	954	Age ≥65 years	TUSCANY, ITALY	Depression- CES-D Vit D- < 50nmol/L	VDD is a risk factor for the development of depression.
Chan et al. (2011) [46]	COHORT	939	Men aged ≥65 years	HONG KONG, CHINA	NA	Inverse relationship exists between serum 25(OH)D and depression
Lee et al. (2011) [47]	COHORT	3369	Men aged 49-71 years	EUROPE	Depression- BDI-II Vit D and PTH levels by radioimmunoassay	Inverse relationship between depression and 25(OH)D levels
Stewart et al. (2010) [48]	NA	2070	Adults ≥ 65 years	ENGLAND	Depression- GDS Vit D levels	Late-life depression is linked to VDD
Cassidy-Bushrow et al. [54] (2012)	NA	178	African- American Pregnant women	DETROIT, USA	Depression- CES-D Vit D levels	Increased depressive symptoms were seen in pregnant women with lower Vit D levels
Brandenbarg et al. [56] (2012)	COHORT	4101	Pregnant women	AMSTERDAM	Depression- CES-D Vit D levels	Increased depressive symptoms were seen in pregnant women with lower Vit D levels
Gur et al. [57] (2014)	COHORT	179	Pregnant women	TURKEY	Depression- EPDS Vit D levels	VDD during pregnancy may have a role in the onset of PPD
Fu et al. [58] (2014)	COHORT	213	Pregnant women	BEIJING, CHINA	Depression- EPDS Vit D levels	PPD associated with low vit D levels

Treatment and management

Even though studies evaluating the relationship between vitamin D and mental well-being use a variety of study groups, outcomes, and behavioral assessments, the findings are consistent, suggesting that vitamin D blood levels or supplementation may positively influence mental health [59]. Based on these observations, it is possible to conclude that vitamin D is essential for mental health, regardless of the examined group or effect linked with mental health [59].

In Adolescents

Högberg et al. concluded a positive association between vitamin D supplementation and improvement of depressive symptoms in depressed adolescents with low serum 25(OH)D in Sweden [60]. Their mean serum 25(OH)D was 41 nmol/L at baseline and 91 nmol/L (p < 0.001) after supplementation, with a significant amelioration of depression according to the Mood and Feelings Questionnaire (MFQ-S) (p < 0.05) [60]. This study is similar to the interventional study done in Iran by Bahrami et al., where for nine weeks, 940 adolescent girls were given vitamin D3 at a level of 50,000 IU/week, and a substantial reduction in depression scores (8 (4-16) vs 7 (2-14)**; **p =.001) after nine weeks of vitamin D treatment along with a dramatic increase in median serum 25(OH)D levels (6.7 ng/mL at baseline vs 35.5 ng/mL after the intervention; p >.001) was noticed [61]. Their results indicate that vitamin D supplementation benefits adolescents with depressive symptoms and low levels of vitamin D at baseline [60,61].

In Adults

Sepehrmanes et al. randomly assigned adults diagnosed with MDD to receive 50,000 IU of Vitamin D (n = 20) or placebo (n = 20) for eight weeks (Table 4) [62]. Improvements in serum 25(OH)D concentrations were considerably greater in the vitamin D group (+20.4 μg/L) than compared to the placebo group (-0.9 μg/L, P < 0.001) after eight weeks of intervention. Also, there was a trend toward a higher decline in the BDI scores in the vitamin D group than in the placebo group [62]. This study can be compared to a cross-sectional and interventional analysis done by Stokes et al. [63], in which 77 depressed patients with chronic liver disease were given 20,000 IUs of Vitamin D for six months (Table 4). In the study's conclusion, it was seen that the severity of depression was inversely related to vitamin D serum levels (β = -0.483, P = 0.004). BDI-II scores also improved significantly from baseline after three and six months (P = 0.003 and P = 0.004, respectively), and vitamin D's antidepressant impact was found to be more prevalent in women in subgroup studies [63].

In Pregnancy

According to Vaziri et al., vitamin D supplementation during pregnancy can help decrease perinatal depression (Table 4) [64]. One hundred sixty-nine pregnant women received 2000 IU of vitamin D3 or a placebo every day from 26 to 28 weeks of pregnancy until delivery. Although both groups had comparable baseline 25(OH)D concentrations, the vitamin D group had a considerably greater 25(OH)D concentration than the control group at childbirth (p 0.001). There was no link between 25(OH)D concentration and depression score at the start (r = 0.13, p = 0.09). While the vitamin D group had a bigger reduction in depression scores than the control group at 38-40 weeks of pregnancy (p = 0.01), the vitamin D group also showed a greater reduction in depression scores at four and eight weeks after birth (p 0.001) [64].

In Conjunction With Other Antidepressants

Vitamin D combined with other antidepressants could be beneficial in managing depression. Khoraminya et al.​​ randomized adults with depression into two groups to receive a combination of 1500 IU Vit D3 and fluoxetine or fluoxetine alone (Table 4)[65]. At the end of eight weeks, the study showed that vitamin D and fluoxetine combination was more effective than fluoxetine alone in managing depression. Mozaffari-Khosrav et al. studied randomized patients (diagnosed with depression and had vitamin D deficiency) to verify if correcting vitamin D deficiency alleviates depressive symptoms (Table 4) [66]. One hundred twenty patients received an intramuscular injection of 150,000 IU vitamin D (n = 40) or 300,000 IU vitamin D (n=40) or nothing (n=40). After three months, it was found that there was a significant difference in mean BDI II test score (P = 0.003) between the group that received 300,000 IU vitamin D injection and the group that received nothing. The study's findings demonstrated that correcting vitamin D deficiency alleviates depressive symptoms and that a single injectable dose of 300,000 IU of vitamin D was both safe and beneficial compared to a 150,000-IU dose [66]. Therefore, management of depression with vitamin D supplementation can significantly impact, as vitamin D is both an effective antidepressant and a cost-efficient option [33]. While those with severely low levels of serum vitamin D would benefit from supplements, people with adequate amounts of serum vitamin D levels will not benefit from it and would not notice a reduction in depression [33].

**Table 4:** Summary of included studies showing the association between vitamin D supplementation and depression RCT- randomised controlled trial, GA- gestational age, MDD - major depressive disorder, VDD- vitamin D deficiency, IU- International units, Vit D- Vitamin D, BDI- Beck depression inventory, EPDS- Edinburgh Postnatal Depression Scale, CRP- C-reactive protein, Ca- Calcium, P- phosphate, PTH- Parathyroid hormone, HDRS- Hamilton Depression Rating Scale, NA- not available

References	Type of study	Sample size	Population	Location	Duration	Intervention	Diagnostic criteria	Conclusion
Sepehrmanesh et al. [62] 2015	RCT	40	Aged between 18-65 years Diagnosed with MDD	NA	8 weeks	50,000 IU vit D per week (n = 20) or placebo (n = 20)	BDI Vit D level Glucose, CRP Lipid profile	improvement of BDI score after Vit D supplementation
Stokes et al. [63] 2016	Cross sectional & Interventional	188	Chronic liver disease patients with depression	GERMANY	6 months	20,000 IU per week	BDI-II Vit D leveL	BDI-II scores improved significantly from baseline after three and six months & Vit D's antidepressant impact was found to be more prevalent in women in subgroup studies
Vaziri et al. [64] 2016	RCT	169	Pregnant women ≥18 years, GA 26-28 week	SHIRAZ, IRAN	From 26-28 week gestation to 8 week postpartum	2000 IU Vit D3 daily from 26 to 28 weeks of gestation until delivery.or placebo	EPDS Vit d level	Use of 2000 IU vitamin D3 per day throughout late pregnancy was found to be beneficial in decreasing perinatal depression.
Khoraminya et al. [65] 2013	RCT	42	Aged between 18-65 years Diagnosed with MDD	TEHRAN	8 weeks	1500 IU VitD3 + 20 mg fluoxetine or fluoxetine alone	HDRS BDI Vit D level	Vit D and fluoxetine combo is more effective than fluoxetine alone.
Mozaffari-Khosrav et al. [66] 2013	RCT	120	Depressed patients with VDD	YAZD, IRAN	3 months	1 single injection of 150,000 IU or 1 single injection of 300,000 IU of vit D or none	BDI-II Vit D level Ca, P, PTH	Correcting vitamin D deficiency alleviates depressive symptoms, and a single injectable dose of 300,000 IU of vit D was both safe and beneficial when compared to a 150,000-IU dose

Limitations

The causes of depression are multifactorial and include multiple genetic, environmental, and social factors, and this article has solely focused on the causes and effects of vitamin D deficiency. Also, this study does not address the effect of vitamin D deficiency in the paediatric population.

## Conclusions

In this article, we discussed depression and determined vitamin D's relationship with depression in its pathogenesis and management across various population groups. We notably discussed how vitamin D deficiency could impact brain structure, function, and effects among adults. The inverse correlation between depression and serum vitamin D levels and the therapeutic benefits of supplementing vitamin D levels highlights the clinical implication of this article. Depressed patients require a tailored approach to treatment, and vitamin D levels should be evaluated as part of their routine assessment. Furthermore, we feel that additional studies need to be conducted in exploring vitamin D's relationship with depression management and universally defining low vitamin D status, thereby formally correlating the two to form an integrated approach to managing depression.
